# Consumer attitudes about the role of multivitamins and other dietary supplements: report of a survey

**DOI:** 10.1186/s12937-015-0053-9

**Published:** 2015-07-02

**Authors:** Annette Dickinson, Douglas MacKay, Andrea Wong

**Affiliations:** 1 Dickinson Consulting, LLC, 3432 Denmark Avenue, #350, 55123 Eagan, MN USA; 2 Scientific and Regulatory Affairs, Council for Responsible Nutrition, 1828 L Street, N.W., Suite 510, 20036 Washington, D.C., USA

**Keywords:** Dietary supplements, Multivitamins, Calcium supplements, Vitamin D supplements, Surveys of dietary supplements, Consumer attitudes, Surveys of consumer attitudes

## Abstract

**Background:**

U.S. nutrition surveys find that intakes of many nutrients fall short of recommendations. The majority of U.S. adults use multivitamins and other dietary supplements as one means of improving nutrient intakes. Some policy makers and health professionals appear reluctant to recommend routine use of dietary supplements to fill nutrient gaps in the diet, in part because they are concerned that people will view the supplements as a substitute for dietary improvement and that the use of supplements may lead to overconsumption of micronutrients. Surveys find that in fact users of dietary supplements tend to have better diets and adopt other healthy habits, suggesting that the supplements are viewed as one aspect of an overall effort to improve wellness. Furthermore, evidence demonstrates that the incidence of excess micronutrient intake is low. We report the results of a survey probing consumer attitudes about the role of dietary supplements.

**Methods:**

The Council for Responsible Nutrition funded a survey to measure consumer attitudes about the role of multivitamins, calcium and/or vitamin D supplements, and other supplements in improving dietary intakes. The research was designed and analyzed by FoodMinds and was fielded using Toluna’s On-line Omnibus. The weighted sample of 2159 respondents is representative of U.S. adults.

**Results:**

Nearly 90 % of the survey respondents agreed that multivitamins and supplements of calcium and/or vitamin D can help meet nutrient needs when desirable intakes are not achieved through food alone. At the same time, 80 % agreed that dietary supplements should not be used to replace healthy dietary or lifestyle habits, and 82 % agreed that people considering taking a high dose, single nutrient supplement should talk with their physician.

**Conclusions:**

These results provide additional support for the conclusion that the vast majority of consumers recognize that multivitamins and other supplements can help fill nutrient gaps but should not be viewed as replacements for a healthy diet. This suggests that policy makers and health professionals could feel comfortable recommending rational dietary supplementation as one means of improving nutrient intakes, without being unduly concerned that such a recommendation would lead consumers to discount the importance of good dietary habits.

## Background

Results from the National Health and Nutrition Examination Surveys (NHANES) document the fact that nutrient intakes of U.S. adults often fall short of target levels [1]. For some nutrients, including calcium and vitamin D, the shortfall is sufficient to be identified as a “public health concern” [2]. Potential means for improving nutrient intake include overall improvement of dietary choices, enrichment or fortification of foods with specific nutrients, and utilization of multivitamins or other dietary supplements to fill nutrient gaps. There is strong support among health professionals and policy makers for efforts to improve dietary choices through the provision of information such as the Dietary Guidelines for Americans and through the provision of financial aid and healthy foods for low-income populations and for people at special nutritional risk including women, infants, children, and the elderly. There is likewise strong support for the selective science-based enrichment or fortification of foods, including the addition of vitamins A and D to milk, the iodization of salt, and the addition of several B vitamins and iron to refined grain products.

Although there is a lack of scientific consensus on whether the routine use of multivitamins by the general population is appropriate, the majority of U.S. consumers (two-thirds or more in some studies) currently use multivitamins and other dietary supplements [3–6]. The Dietary Reference Intakes and the 2010 Dietary Guidelines for Americans recommend routine supplemental intakes of some nutrients for some population groups, including folic acid for women of childbearing age, iron for pregnant women, and vitamin B-12 for adults over 50 years of age [2, 7]. Some prominent scientists recommend routine use of multivitamins for most adults, to fill known nutritional gaps, ensure normal body function, and generally support good health -- effects which may also provide some protection against chronic disease [8–10].

Some scientists oppose routine use of multivitamins, largely on the grounds that multivitamins have not been shown to provide substantial protection against chronic diseases such as cancer and heart disease [11]. Others believe that the use of a daily multivitamin should be carefully considered for most middle-aged and older individuals to not only ensure adequate dietary intake of essential vitamins and minerals, but also potentially reduce the risk of total cancer without any evidence of harm [12]. However, it should be recognized that disease prevention is not the rationale for the existence of multivitamins and is not the primary reason consumers give for using multivitamins and other dietary supplements [6, 13]. Naturally, people have some interest in disease prevention, but the top reason given by consumers for using dietary supplements is for overall health and wellness.

The 2010 Dietary Guidelines for Americans did not recommend routine use of multivitamins or of recognized shortfall nutrients such as calcium and vitamin D for the general population, in part because of concern that dietary supplements might be viewed by consumers as a substitute for dietary improvement [2]. Surveys have repeatedly shown, on the contrary, that the use of dietary supplements is associated with better diets and the adoption of other healthy lifestyle habits, suggesting that consumers understand dietary supplements to be just one component of a larger effort to improve both dietary intake and overall health [3–6].

To better characterize consumer attitudes regarding the role that multivitamins, supplements of calcium and/or vitamin D, and other dietary supplements may play as aids to safely improving dietary intake, the Council for Responsible Nutrition (CRN), a trade association representing the dietary supplement industry, sponsored a consumer attitude survey in October 2014. We report the results of that survey.

## Methods

The Council for Responsible Nutrition sponsored a survey to measure consumer comprehension of statements regarding the use of multivitamins or dietary supplements of calcium and/or vitamin D, and to measure consumer attitudes regarding the role of multivitamins and other dietary supplements as aids to improving overall dietary intake or health status. The research was designed and analyzed by FoodMinds and was fielded October 10–14, 2014 using Toluna’s On-line Omnibus.

FoodMinds is a food and nutrition communications consulting company, specializing in advising clients on issues relating to food, nutrition, health, and wellness. Toluna is an internationally recognized provider of online panel and survey technology, providing clients with access to 41 actively managed panels, globally. Toluna actively recruits people to register as members of an on-line panel and verifies the identity and reliability of panel members. Toluna’s On-line Omnibus panel in the U.S. has about 1.5 million members who can potentially respond to an invitation to participate in a survey for which they are eligible. Incentives are provided to encourage panel members to participate in surveys, in the form of points which can be redeemed for cash, gift vouchers, or other rewards.

Survey quotas for the base sample were established based on census data for age, gender, and region. Eligible panel members were invited to participate, but were not informed in advance of the topic of the survey. When the survey’s quotas were fulfilled for total participants or for ratios established for gender, age, or region, the survey was closed to additional participants. Responses were weighted where necessary to bring them into line with actual proportions in the population for age, gender, race/ethnicity, education, region, and household income. The base sample included 2159 adults age 18 and over.

Respondents in the CRN consumer attitude survey were asked to rate their perception of two statements, rating four aspects of each statement on a seven-point scale. The four aspects rated were whether the statements were easy or difficult to understand, whether the statements were or were not relevant to the respondent, whether the statements did or did not provide new information, and whether the information was or was not considered to be an important reminder for the respondent’s personal health. The first statement was: “To ensure nutrient adequacy, people may consider taking a multivitamin and mineral supplement when recommended vitamin and mineral intake cannot be met through food alone.” The second statement was: “Americans who do not consume the recommended amounts of calcium and vitamin D through food sources should consider a calcium and vitamin D supplement to help support bone health.”

Respondents were then asked to indicate their level of agreement or disagreement with each of seven statements, on a five-point scale ranging from “strongly agree” to “strongly disagree.” The possible responses on the five-point scale included “strongly agree,” “agree,” “neither agree nor disagree,” “disagree,” and “strongly disagree.” The seven statements suggest that multivitamins can help fill nutrient gaps in the diet, that supplements of calcium and/or vitamin D can support bone health when the diet does not provide adequate amounts of these nutrients, that multivitamins are just one part of a healthy diet and should not be used to replace healthful dietary and lifestyle habits, that multivitamins are not medicine and are not meant to cure diseases, and that people should talk with their physician if they are considering taking a high dose single nutrient supplement. See Table 1 for wording of each statement.

**Table 1:** Percent of survey respondents who agreed or disagreed with each of seven statements about the role of multivitamins and other dietary supplements in helping to improve dietary intake and health

Agree or strongly agree	Statement	Neither agree nor disagree	Disagree or strongly disagree
88 %	Calcium and D supplements can help support bone health when people do not consume recommended amounts of calcium and D from food sources*	11 %	2 %
87 %	MVM can help people meet nutrient needs that can’t be met through food alone	11 %	2 %
82 %	People considering taking a high dose, single nutrient supplement should talk with their physician	15 %	3 %
81 %	MVM should be considered as just one part of a healthy diet	15 %	4 %
80 %	MVM should not be used to replace healthful dietary and lifestyle habits	15 %	5 %
75 %	MVM are not meant to cure diseases	19 %	6 %
67 %	MVM are not medicine*	24 %	10 %

*Responses to these two questions do not add to exactly 100 % because of rounding

*MVM* multivitamin and mineral supplements

## Results

The vast majority of the 2159 respondents perceived the two basic statements about multivitamins and about calcium and/or vitamin D supplements to be easy to understand -- 84 % found the multivitamin statement easy to understand, and 88 % found the calcium/D statement to be easy to understand (rating of 5, 6, or 7 on a seven-point scale).

About three-quarters of the respondents found both statements to be relevant to them and also to be important reminders for their own health -- 73 % found the multivitamin statement to be relevant and important, 74 % found the calcium/D statement to be relevant, and 75 % found the calcium/D statement to be an important reminder for their own health. However, only about half the respondents perceived the information in either statement to be “new” -- 46 % said the multivitamin statement provided new information, and 50 % said the calcium/D statement provided new information.

When asked whether they agreed or disagreed (on a five-point scale from “strongly agree” to “strongly disagree”) with each of seven general statements about the role of multivitamins or supplements of calcium and/or vitamin D in the context of a healthy diet and lifestyle, 67 % to 88 % of the respondents agreed or strongly agreed with each statement. For each of the seven statements, the proportion who “strongly agreed” (36 to 54 %) was greater than the proportion who simply “agreed” (28 to 39 %). See Table 1 for the text of the seven statements and the percentage of respondents agreeing or disagreeing with each statement.

Almost 90 % of respondents agreed that calcium and vitamin D supplements can help support bone health when dietary intake is not sufficient (88 %) and that multivitamin and mineral supplements can help meet nutrient needs when people don’t get enough from food alone (87 %). About 80 % agreed that people should talk with their physician before using a high dose single nutrient supplement (82 %), that a multivitamin should be considered as just one part of a healthy diet (81 %), and that a multivitamin should not be used to replace healthy diet and lifestyle habits (80 %). Three-fourths of respondents agreed that multivitamins are not intended to cure disease, and two-thirds agreed that multivitamins are not medicine.

## Discussion

The results of the CRN consumer attitude survey suggest that consumers understand the supportive role dietary supplements can play in helping to ensure adequate nutrient intake and also understand that dietary supplements do not substitute for a good diet or a healthy lifestyle. Furthermore, the survey results suggest that consumers are aware that they should avoid over-consuming micronutrients by talking with their doctor if they are considering use of high dose single nutrient supplements.

The shortfalls in nutrient intake identified by large national surveys are widespread and are not small in magnitude. In the 2003–2006 National Health and Nutrition Examination Survey (NHANES), 93 % of respondents ages 2 years and older had intakes of vitamin D from food sources (naturally occurring and enriched/fortified foods) that were below the Estimated Average Requirement (EAR); 90 % were below the EAR for vitamin E; 45 % were below the EAR for vitamin A; 37 % were below the EAR for vitamin C, and 49 % were below the EAR for calcium [1]. These low intakes need to be raised, and when the contributions from dietary supplements were considered they were in fact raised. In the same NHANES survey, the percentage of respondents who did not meet the EAR decreased by 11 to 30 % for the aforementioned micronutrients when all sources, including dietary supplements, were considered, in comparison to food sources only [1]. These data demonstrate the efficacy of dietary supplement use in filling nutrient gaps. The CRN consumer attitude survey confirms that people understand that multivitamins and supplements of calcium and/or vitamin D can help fill nutrient gaps in the diet.

The NHANES 2003–2006 data also demonstrate that nutrient intake from fortification and dietary supplements does not present a meaningful risk for overconsumption. The percentage of the population with total intakes of most micronutrients greater than their respective Tolerable Upper Intake Levels (ULs) was very low. Combined intakes from foods, fortification, and supplementation resulted in less than 1 % of the population exceeding the UL for vitamin C, D, or E, while less than 3 % exceeded the UL for calcium [1]. For vitamin A and folate, 1 or 2 % of the population exceeded the UL based on intakes from food and fortification alone, and the addition of supplementation increased this fraction to 5 or 6 % [1]. The authors of this report note that the UL is defined as “the highest level of daily intake that is likely to pose no risk” and point out that “more research is needed on the adverse health effect, if any, from intake levels exceeding the UL” [1].

Long-term clinical trials support the safety of daily MVM supplementation. In the recent Physicians’ Health Study II, a trial of over 14,000 male physicians that took a daily MVM for over ten years, no serious adverse effects were found [12]. Furthermore, a 2013 systematic review of available scientific evidence showed that supplementation with a MVM does not increase all-cause mortality, cancer incidence or mortality, or CVD incidence or mortality [14].

Use of multivitamins and other dietary supplements is prevalent among U.S. consumers and is associated with somewhat better nutrient intakes from diet alone and also with the adoption of other healthy lifestyle habits. Adult use of dietary supplements in NHANES 2003–2006 was 54 % overall but increased with age and was 58 % and 72 % in men and women, respectively, in the age range 51–70 [3]. The Multiethnic Cohort Study also reported 58 % use in men and 72 % use in women in a sample of more than 100,000 healthy adults over the age of 45 [5]. Interestingly, surveys show that dietary supplement use is just as prevalent among dietitians and other health professionals, including doctors and nurses, as among the general population [6, 15]. Users of dietary supplements tend to adopt other healthy habits including exercising, not smoking, avoiding obesity, and consuming somewhat better diets, leading the authors of the report on the Multiethnic Cohort to conclude that these findings “suggest that a ‘health conscious’ attitude predominates among dietary supplement users” [5]. The CRN consumer attitude survey confirms that people understand that, while multivitamins and other dietary supplements can be beneficial in providing additional intakes of shortfall nutrients, they do not substitute for overall dietary improvement or the adoption of other healthy lifestyle habits. The CRN consumer attitude survey also indicates that people know they should talk to their doctor if they are considering using high dose, single nutrient supplements.

The top reasons given by consumers for using multivitamins and other dietary supplements focus on overall wellness. In NHANES 2007–2010, the most prevalent reasons given for using dietary supplements were to improve overall health (45 %) and to maintain health (33 %) [13]. In a 2011 CRN consumer survey and a 2009 CRN survey of dietitians, the most prevalent reason given by consumers for using dietary supplements was overall health and wellness (58 %), while for dietitians overall health and wellness ranked second (53 %) behind bone health (58 %) [6, 15]. Consumers are, of course, also interested in disease prevention, but it is not the primary motivation given for using dietary supplements. The CRN consumer attitude survey confirms that people understand that multivitamins are not meant to cure disease or to be used as medicines.

The current survey data provide insight on consumer attitudes in the general adult population. However, a limitation of the survey is that it does not distinguish between the perceptions of dietary supplement users and those of non-users and does not stratify responses by demographic factors such as age. Future investigation into the potential variations in attitudes by age, gender, or other demographic factors may be desirable.

In the CRN consumer attitude survey reported here, respondents expressed perceptions consistent with the available evidence regarding the habits and motivations of dietary supplement users. They recognize the benefits of multivitamins and of calcium and/or vitamin D supplements in filling nutrient gaps and therefore improving health, but they also recognize that the use of dietary supplements does not replace the need to focus on overall dietary improvement and to adopt other healthy lifestyle habits.

## Conclusions

These results provide additional support for the conclusion that the vast majority of consumers recognize that multivitamins and other supplements can be helpful in filling nutrient gaps in the diet but should not be viewed as replacements for a healthy diet or healthy lifestyle habits. This suggests that policy makers and health professionals could feel comfortable recommending rational dietary supplementation as one means of improving nutrient intakes, without being unduly concerned that such a recommendation would lead consumers to discount the importance of good dietary habits. A large majority of consumers already use multivitamins and other dietary supplements, and that use has been shown to decrease the prevalence of nutrient shortfalls. Users of dietary supplements deserve the support of policy makers, to the extent that their choice is a logical and rational one.

## Abbreviations

CRN: Council for Responsible Nutrition
DGA: Dietary Guidelines for Americans
DRI: Dietary Reference Intakes
MVM: Multivitamin and mineral dietary supplements
NHANES: National Health and Nutrition Examination Survey
